# Diagnostic Study to Assess the Performance of a New Urinary *Legionella* Antigen Test—A National Study in Three Referral University Hospitals in Austria during 2014–2017

**DOI:** 10.3390/ijerph192416705

**Published:** 2022-12-13

**Authors:** Ziad El-Khatib, Lukas Richter, Jutta Ressler, Bernhard Benka

**Affiliations:** 1 Institute for Surveillance & Infectious Disease Epidemiology, Austrian Agency for Health and Food Safety (AGES), Währingerstrasse 25a, 1090 Vienna, Austria; 2 Department of Global Public Health, Karolinska Institutet, 17177 Stockholm, Sweden; 3 Institute for Medical Microbiology and Hygiene, Austrian Agency for Health and Food Safety (AGES), Währingerstrasse 25a, 1090 Vienna, Austria

**Keywords:** Legionella, rapid testing, Austria, ELISA

## Abstract

Background: We evaluated the performance of a rapid diagnostic antigen test (Coris) as an index test versus the urinary Antigen ELISA (Bartels) as the reference test. Methods: Prospective diagnostic accuracy study (2014–2017) at three university hospitals in Austria. Results: A total of 996 patients were included in the study. Legionellosis was diagnosed in 49/996 (4.9%) using the reference test. The sensitivity and specificity of the Coris test were 75.5% (95% CI 61.1–86.7%) and 100% (95%CI 99.6–100%), respectively. The PPV was 100% and when using the lower 95% CI limit of the estimate for sensitivity, the resulting PPV was 61.1%. The NPV was 98.7% and the accuracy was 98.8%. The index test showed a PPV > 97% during the period of summer and autumn (May through November) and ≥88% during winter (December through February). The NPV was >97% during all of the periods. The median of the monthly incidence in the general population was 0.1 per 100,000 (IQR 0.1; 0.3). Conclusion: The new rapid test gave a high level of diagnostic accuracy in a rapid fashion. The test can be applied at the bedside by non-laboratory staff.

## 1. Introduction

Community-acquired pneumonia (CAP) is a leading cause of hospitalization worldwide, with a mortality rate ranging between 8–30% among hospitalized patients [1]. Patients with CAP are at a high risk of morbidity, compromised quality of life, and mortality. Approximately up to 25% of CAP patients require treatment [2]. The *Legionella* species is one of the attributable risks (2–8%) of pneumonia—and it is unknown in 30–60% of the cases [2]. Legionellosis has two types of clinical presentations, including mild upper respiratory infection with non-specific influenza-like illness symptoms (Pontiac fever (PF)) and Legionnaire’s disease (LD), with more severe clinical presentation including pneumonia. The detection of *Legionella* using radiography and regular laboratory diagnostics is not a reliable method. Additionally, the risk of mortality is higher among patients admitted to intensive care units, older patients, and among patients that have received an incomplete antibiotic therapy [3]. Therefore, accurate diagnostic methods are required to screen *Legionella*, in order to provide the correct antibiotic therapy in a timely manner [4]. The use of urine antigen tests increases the chances of the early diagnostics for Legionnaires’ disease, which decreases the risk of mortality. Currently, *Legionella* is screened, in urine, using Enzyme-Linked Immunosorbent Assay (ELISA), which is known to be timely, sensitive, and specific in the diagnosis of Legionellosis [5]. The Legionella K-SeT (Coris BioConcept, Gembloux, Belgium) is a rapid immunochromatographic assay (ICT). It detects soluble antigens from *Legionella pneumophila* serogroup 1 (Lp1) in urine. In general, the Lp1 antigen is detectable in urine post two days of the onset of clinical symptoms, and it can persist for over two months after the clearance of disease [6]. 

Briefly, this is a ready-to-use membrane test based on colloidal gold particles. It allows for the detection of *Legionella pneumophila* lipopolysaccharide in urine samples. Legionella K-SeT sensitivity and specificity come from monoclonal and polyclonal anti-Legionella antibodies. Some antibodies are conjugated to colloidal gold particles and dried on a conjugate absorbent pad. Each strip is sensitized with anti-Legionella antibodies at the upper line and with a control antibody at the bottom (migration control) line. The whole procedure is completed within 15 min and it is considered to be a more technically optimized method (less complex and requires less equipment) than ELISA. It allows for the identification of a wide *Legionella* urinary antigen by conventional laboratories. Epidemiologically, the *Legionella*-associated CAP has a seasonal-associated feature; more cases are reported during the summer [7,8]. However, hospital-associated *Legionella* cases are not associated with seasonality [7,8]. In this study, we assessed the Legionella K-SeT test at three medical university hospitals in Austria. To determine its sensitivity and specificity, we compared this assay with the ELISA (Bartels, Trinity, Biotech, Ireland) diagnostic standard procedures.

## 2. Materials and Methods 

### 2.1. Study Design, Study Setting, and Study Participants

We applied a diagnostic accuracy study for a consecutive series of individuals in whom the target condition—Legionella pneumonia—was suspected. We used a one-gate study design; this uses a single source of study participants. All of the patients had clinically and radiologically confirmed pneumonia, and were suspected to have the target condition, Legionella pneumonia. All of the study participants underwent both tests, starting with the index test followed by the reference standard. The accuracy outcomes of the index test were determined based on the results obtained from the standard reference [9,10]. 

*Inclusion criteria*: In-hospital patients, with clinical or radiological signs compatible with pneumonia, admitted at the University Hospital, Innsbruck; University Hospital, Graz; and University Hospital, Salzburg during the period of August 2014 until November 2017.

### 2.2. Recruitment of the Study Participants and Collection of Urine Specimens

The recruitment of the study participants was done according to the 10-year average monthly incidence of *Legionellosis* in order to match the seasonality of *Legionellosis* (see inclusion list of patient number/week below). Every site included the first occurring pneumonia patients according to the requested weekly number. Urine specimens from the recruited patients were stored in standard sterile containers at −20 °C and sent in leak-proof containers with dry ice to the National Reference Laboratory in Vienna.

### 2.3. Test Methods

#### Index Test and Standard Reference

The **index test** was the Legionella *K*-SeT (CORIS BioConcept, Gembloux, Belgium), an immunochromatographic assay. The diagnostic threshold was defined as the appearance of a test line. The **reference standard test**, used routinely in practice for confirming an infection with *Legionella* pneumophila, was ELISA Bartels Legionella Urinary Antigen (LUA) (Legionella Urinary Antigen, Intracel, Issaquah, WA, USA). It is an enzyme-linked immunosorbent assay detecting soluble urinary antigen of *L. pneumophila* sg1. The diagnostic threshold of the reference standard test was defined as four times the mean optic density value of the negative control (1–9). The urine samples were kept at room temperature, prior to the application of these two tests. No clinical information was available for the performers of the index and reference standard tests. The reference tests were performed at the hospital and the staff may have been exposed to the clinical information of the patients.

### 2.4. Analysis

#### 2.4.1. Sample Size Calculation

The sample size calculation was performed using the formula: $N_{SRS}=\frac{Se\;\left(1-Se\right)}{L^{2}p}\;z_{\;1-\alpha /2}^{2}$. *L* is the desired width of one-half of the confidence interval, *p* is the conjectured prevalence of the Legionnaire, and *Se* is the conjectured sensitivity of the index test.

The estimated study sample size was between 984 and 1009 patients; this was based on a *z*-value of 1.96, *L*-value of 8% (7%), and a conjectured prevalence of Legionnaires’ disease among patients with pneumonia of 5% and a conjectured sensitivity of the index test of 91%.

#### 2.4.2. Statistical Analysis 

The outcome measures were the sensitivity and specificity of the index test, provided with the 95% confidence interval. The formulas used were $\frac{\mathrm{TP}\;\times 100}{\left(\mathrm{TP}\;+\;\mathrm{FN}\right)}$ and $\frac{\mathrm{TN}\;\times 100}{\left(\mathrm{TP}\;+\;\mathrm{FP}\right)}$, respectively [9,10].

For the accuracy, we used the formula: $\frac{\left(\mathrm{TP}\;+\;\mathrm{TN}\right)}{\left(\mathrm{TP}\;+\;\mathrm{TN}\;+\;\mathrm{FP}\;+\;\mathrm{FN}\right)}$.

For calculating the overall and monthly PPV and NPV, we used the sensitivity and specificity estimates of the index test, and the prevalence of Legionnaires’ disease among pneumonia patients, defined as the proportion of study participants, who tested positive for the urinary Legionella antigen using the reference test. We used the formulas below.

$$\mathrm{PPV}=\frac{\left(\mathrm{Sensitivity}\;\times \;\mathrm{Prevalence}\right)}{\left(\left(\mathrm{Sensitivity}\;\times \;\mathrm{Prevalence}\right)+\left(1-\mathrm{Specificity}\right)\times \left(1-\mathrm{Prevalence}\right)\right)}$$

$$\mathrm{NPV}=\frac{\left(\mathrm{Specificity}\;\times \left(1-\;\mathrm{Prevalence}\right)\right)}{\left(\left(1-\;\mathrm{Sensitivity}\right)\times \;\mathrm{Prevalence}\;+\;\mathrm{Specificity}\;\times \left(1-\;\mathrm{Prevalence}\right)\right)}$$

The 95%CI was calculated using the following formula: sensitivity ±1.96 (SE sensitivity) and specificity ±1.96 (SE specificity). Finally, we plotted the PPV against the monthly prevalence with an overlaid prediction plot. We used Stata/SE 13.1 for the latest analysis (command *twoway scatter fpfit*). We conducted the analysis of NPV using two different methods: First, according to the suggestion of Black and Armstrong [11], we used for calculation of the NPV, for the specificity 99.9% instead of 100% and second, we used the lower limit for 95%CI of sensitivity and specificity. 

## 3. Results

During the three year study period (2014–2017), a total of 996 patients were included as study participants. The urine specimens for *Legionella* testing were available. *Legionellosis* was identified in 49 of the 996 (4.9%) study patients through detecting the legionella urinary antigen using the reference test, ELISA (Table 1). 

Of the 49 *Legionella* antigen positive urinary specimens, 37 tested positive with the index test, which resulted in a sensitivity of 75.5% (95%CI 61.1–86.7%). All 947 urinary specimens, which tested negative for the *Legionella* antigen using the reference test, also tested negative by the index test, resulting in a specificity of 100% (95%CI 99.6–100%). The overall PPV was 100%. Using the arbitrary value for specificity of 99.9% (instead of 100%), the resulting PPV was 97.5% and the NPV was 98.7% (see method 1 in Table 2). When it came to using the lower 95%CI limit of the estimate for the sensitivity (i.e., 61.1%), the resulting PPV was 88.7% and the NPV was 98.0% (see method 2 in Table 2). The index test showed a PPV > 97% during the period of May through November and ≥88% in December and February. When it came to NPV, it was >97% during all months (Table 3). The median of the monthly incidence in the general population was 0.1 per 100,000 (IQR 0.1; 0.3) (see Table 3). The trend Legionella test method for reference for prevalence and PPV showed a coefficient of 9.5 (R^2^ 0.58, *p* < 0.01).

## 4. Discussion

The rapid urinary antigen testing for the *Legionella* antigen has been a useful method for LD diagnosis. In this prospective study, with a representative sample of the general population, we compared two *Legionella* tests during a period of three years. Legionella was identified in 5.1% of the patients diagnosed at three large medical university hospitals in Austria. Legionella is a seasonal disease, so its prevalence peaks towards the summer [12,13], and a systematic review by Shimada et al. reported that *Legionella* tends to be identified among older men [14]. Due to the seasonality of *Legionella,* we designed our study to deal with this range of prevalence; for example, we calculated the PPV and NPV according to the prevalence throughout the year. The NPV value remained high despite the changes in the prevalence, which is important clinically, so as to ensure the absence of *Legionella*. In addition, a rapid test confirming positive urinary antigen test can prompt the withdrawal of antibiotic treatment directed to non-legionella pathogens [15]. Although we did not compare the rapid test to the culture testing, it is worth mentioning that the sensitivity result was higher than the culture testing, which was considered between 20% and 80% [16].

To the best of our knowledge, this was the first study to assess this index rapid test in a real-world condition. We conducted the study prospectively, using single-gate study design, which is one of the most appropriate methods to assess diagnostics accuracy [10,17]. The tests methods were used consistently during the study period, which helped us to conduct a comprehensive comparison between these two diagnostics. In addition to the rapid utility of the index testing, this was found to be a quick way to screen for LD (15 min) by health staff that do not have technical training for LD. Additionally, nurses can use it in patients’ care during outbreaks in healthcare facilities (for example in elderly care facilities, or pharmacies during outbreaks, where they are licensed to do it) [18]. Therefore, this index test can be used as a supplement for routine laboratory testing [19]. Traditionally, the diagnosis of LD is conducted using different methods (including special media, processing, and sequencing based typing) and technical expertise [20,21]. The serological testing for *Legionella* has little impact on clinical practice, because approximately one quarter of LD patients do not develop a detectable antibody response when tested too early [20]. Using UA detection of the *L. pneumophila* serogroup has been the most commonly used method for LD diagnosis in clinical settings [20,22]. We achieved a good diagnostic performance for *Legionella*, finding good sensitivity, high specificity, PPV, and NPV values. The index test is useful for diagnosing *Legionella,* especially when the results are obtained in 15–20 min. Time to detection is considered critical for disease management, especially for at-risk populations [23], instead of waiting for up to six days with the urine antigen laboratory testing [15]. In addition, this test does not require highly trained health staff to perform it. The sensitivity of the rapid test was 75.5%, which might be an issue for the efficiency of the test; however, its high level of specificity, accuracy, NPV, and PPV would serve as a way to help diagnose suspected *Legionella* patients. Further studies might be needed to improve the sensitivity of this index test. When it comes to the study limitations, the current study arrangement did not allow us to have a conclusion on NPV. We should acknowledge that there are other methods besides ELISA reported in the literature (13) that we could not assess. Inaddition, Austria has over 90% of *Legionella pneuophilia* that belong to the category Lp 1 [24], so we may have missed a small proportion of patients falling under other serogroups. Additionally, we should note that the clinical sensitivity of urine-based antigen testing is known to be associated with the severity of the *Legionella* disease [25]; therefore, hospitalized patients tend to more likely test positive than patients with mild illness. This means the sensitivity results may not be the same, should the same study be repeated in the general community [26]. The utility of urine is useful as a medium that is obtained by a non-invasive collection method. However, some factors may influence the level of antibodies in urine and eventually the results of ELISA testing. This includes the waiting time required to transport the urine samples and process them, and the temperature [27]. We conducted sample collection according to the incidence distribution of *Legionella*, where it tends to increase during the summer season; however, the prevalence of pneumonia tends to increase during the winter season. In addition, we did not test for the presence of different bacterial contaminants, which may have contributed to the false negative ELISA outcome. The sample size of the confirmed positive cases by both of the index and reference tests was relatively small, which may have affected the sensitivity results. Finally, men tend to be at a higher risk to acquire *Legionella*, but we did not have data on gender so we could not compare the screening between men and women. We should note that the index test did have a high PPV, when the prevalence was high, so we think these results are generalizable. In Austria, all patients presenting with pneumonia symptoms are tested for LD and this would exclude the possibility that LD patients may not be screened.

## 5. Conclusions

In summary, this index test evaluated here was a rapid test that helped with identifying LD and guiding the immediate patients’ management, with a high level of sensitivity and specificity. This test was useful for the rapid diagnosis of *Legionella* pneumonia, by being a good substitute for laboratory-based testing.

## Figures and Tables

**Table 1:** Comparison of the *Legionella* test method results.

		ELISA (Reference)		
		Disease	Non-Disease	Total
		n = 49	n = 947	N = 996
**Index test**	**Positive**	37 (100%)	0	37
	**Negative**	12 (1.2%)	947 (98.8%)	959

**Table 2:** Estimated sensitivity and specificity based on the original analysis and alternative methods explored.

Measurement	Original Analysis	Method 1 *	Method 2 **
**Prevalence**	4.9	4.9	4.9
**Sensitivity**	75.5 (61.1–86.7)	75.5	61.1
**Specificity**	100 (99.6–100)	99.9	99.6
**PPV**	100	97.5	88.7
**NPV**	98.7 (98.0–99.2)	98.7	98.0

* we have adopted an arbitrary value for specificity of 99.9 instead of 100; ** we have adopted a lower limit values for 95%CI of sensitivity and specificity.

**Table 3:** The trend *Legionella* test method for reference for prevalence, PPV, and NPV.

			Reference Test (Bartels)		
Month	Prevalence (%) among Patients Using Index Test (Coris Test)	Prevalence (%) among Patients Using Reference Test (Bartels ELISA)	PPV	NPV *	Average Monthly Incidence per 100,000 Person-Years, 2014–2017
January	0/51 (0%)	0/51 (0%)	0	100	0.1
February	1/54 (1.8%)	1/54 (1.8%)	93.3	99.6	0.1
March	0/43 (0%)	0/43 (0%)	0	100	0.1
April	0/71 (0%)	0/71 (0%)	0	100	0.0
May	5/60 (8.3%)	6/60 (10.0%)	98.8	97.3	0.1
June	4/79 (5.1%)	6/79 (7.6%)	98.4	98.0	0.2
July	4/156 (2.6%)	7/156 (4.5%)	97.3	98.9	0.2
August	8/147 (5.4%)	11/147 (7.5%)	98.4	98.1	0.3
September	6/127 (4.7%)	7/127 (5.5%)	97.8	98.6	0.3
October	5/96 (5.2%)	6/96 (6.2%)	98.0	98.4	0.2
November	3/63 (4.8%)	4/63 (6.3%)	98.1	98.4	0.1
December	1/49 (2.0%)	1/49 (2.0%)	88.4	99.8	0.1

* We used specificity level of 99.9% instead of 100%.

## Data Availability

Not applicable.

## References

[1] Viasus D., Calatayud L., McBrown M.V., Ardanuy C., Carratalà J. (2019). Urinary antigen testing in community-acquired pneumonia in adults: An update. Expert Rev. Anti-Infect. Ther..

[2] Mandell L. (2015). Community-acquired pneumonia: An overview. Postgr. Med..

[3] Ewig S., Torres A. (2011). Community-acquired pneumonia as an emergency: Time for an aggressive intervention to lower mortality. Eur. Respir. J..

[4] World Health Organization (2007). Legionella and the Prevention of Legionellosis. Emerg. Infect. Dis..

[5] Coris BioConcept (2019). Legionella K-SeT. http://catalogue.bioconnections.net/files/K1515_Legionella_cassette_IFU.pdf.

[6] Mercante J.W., Winchell J.M. (2015). Current and emerging legionella diagnostics for laboratory and outbreak investigations. Clin. Microbiol. Rev..

[7] Fields B.S., Benson R.F., Besser R.E. (2002). Legionella and legionnaires’ disease: 25 Years of investigation. Clin. Microbiol. Rev..

[8] Simmering J.E., Polgreen L.A., Hornick D.B., Sewell D.K., Polgreen P.M. (2017). Weather-dependent risk for legionnaires’ disease, United States. Emerg. Infect. Dis..

[9] Buehler A.M., de Oliveira Ascef B., de Oliveira Júnior H.A., Ferri C.P., Fernandes J.G. (2019). Rational use of diagnostic tests for clinical decision making. Rev. Assoc. Med. Bras..

[10] Rutjes A.W.S., Reitsma J.B., Vandenbroucke J.P., Glas A.S., Bossuyt P.M.M. (2005). Case-control and two-gate designs in diagnostic accuracy studies. Clin. Chem..

[11] Black W.C., Armstrong P. (1986). Communicating the significance of radiologic test results: The likelihood ratio. Am. J. Roentgenol..

[12] Ng V., Tang P., Jamieson F., Guyard C., Low D.E., Fisman D.N. (2009). Laboratory-based evaluation of legionellosis epidemiology in Ontario, Canada, 1978 to 2006. BMC Infect. Dis..

[13] Peci A., Winter A.L., Gubbay J.B. (2016). Evaluation and Comparison of Multiple Test Methods, Including Real-time, PCR, for Legionella Detection in Clinical Specimens. Front. Public Heal..

[14] Shimada T., Noguchi Y., Jackson J.L., Miyashita J., Hayashino Y., Kamiya T., Fukuhara S., Matsumura T., Yamazaki S. (2009). Systematic review and metaanalysis: Urinary antigen tests for legionellosis. Chest.

[15] Garbino J., Bornand J.E., Uckay I., Fonseca S., Sax H. (2004). Impact of positive legionella urinary antigen test on patient management and improvement of antibiotic use. J. Clin. Pathol..

[16] Centre for Disease Control and Prevention (CDC) (2020). Legionella: Diagnosis, Treatment, and Prevention.

[17] Leeflang M.M.G., Allerberger F. (2019). How to: Evaluate a diagnostic test. Clin. Microbiol. Infect..

[18] Dwyer D.E., Sintchenko V. (2007). Point-of-care testing for community-acquired pneumonia: Do we have all the answers?. Med. J. Aust..

[19] Miyashita N., Higa F., Aoki Y., Kikuchi T., Seki M., Tateda K., Maki N., Uchino K., Ogasawara K., Kiyota H. (2017). Clinical presentation of Legionella pneumonia: Evaluation of clinical scoring systems and therapeutic efficacy. J. Infect. Chemother..

[20] Avni T., Bieber A., Green H., Steinmetz T., Leibovici L., Paul M. (2016). Diagnostic accuracy of, P.C.R alone and compared to urinary antigen testing for detection of Legionella spp.: A systematic review. J. Clin. Microbiol..

[21] Moran-Gilad J., Prior K., Yakunin E., Harrison T.G., Underwood A., Lazarovitch T., Valinsky L., Lueck C., Krux F., Agmon V. (2015). Design and application of a core genome multilocus sequence typing scheme for investigation of Legionnaires’ disease incidents. Eurosurveillance.

[22] Wever P.C., Yzerman E.P.F., Kuijper E.J., Speelman P., Dankert J. (2000). Rapid diagnosis of Legionnaires’ disease using an immunochromatographic assay for Legionella pneumophila serogroup 1 antigen in urine during an outbreak in The Netherlands. J. Clin. Microbiol..

[23] Mondino S., Schmidt S., Rolando M., Escoll P., Gomez-Valero L., Buchrieser C. (2020). Legionnaires’ Disease: State of the Art Knowledge of Pathogenesis Mechanisms of Legionella. Annu. Rev. Pathol. Mech. Dis..

[24] Austrian Agency for Health and Food Safety (AGES) (2018). Nationale Referenzzentrale für Legionella-Infektionen.

[25] Ferreira-Coimbra J., Rebelo S., Cunha A.L., Guimarães J.T., Bettencourt P. (2019). Legionella urinary antigen test –a contribute towards a more rational use. Eur. J. Intern. Med..

[26] Couturier M.R., Graf E.H., Griffin A.T. (2014). Urine antigen tests for the diagnosis of respiratory infections: Legionellosis, histoplasmosis, pneumococcal pneumonia. Clin. Lab. Med..

[27] Eric Thomas C., Sexton W., Benson K., Sutphen R., Koomen J. (2010). Urine collection and processing for protein biomarker discovery and quantification. Cancer Epidemiol. Biomark. Prev..

